# Diet Quality and Bone Density in Youth with Healthy Weight, Obesity, and Type 2 Diabetes

**DOI:** 10.3390/nu13093288

**Published:** 2021-09-21

**Authors:** Joseph M. Kindler, Sina Gallo, Philip R. Khoury, Elaine M. Urbina, Babette S. Zemel

**Affiliations:** 1 Department of Nutritional Sciences, The University of Georgia, Athens, GA 30602, USA; sina.gallo@uga.edu; 2 Division of Gastroenterology, Hepatology and Nutrition, The Children’s Hospital of Philadelphia, Philadelphia, PA 9146, USA; zemel@chop.edu; 3 The Heart Institute, Cincinnati Children’s Hospital Medical Center, Cincinnati, OH 45229, USA; phil.khoury@cchmc.org (P.R.K.); elaine.urbina@cchmc.org (E.M.U.); 4 Department of Pediatrics, University of Cincinnati, Cincinnati, OH 45229, USA; 5 Department of Pediatrics, The University of Pennsylvania, Philadelphia, PA 9146, USA

**Keywords:** bone density, type 2 diabetes, youth, diet, obesity

## Abstract

Purpose: To assess relationships between diet quality and areal bone mineral density (aBMD) in youth with healthy weight, obesity, and type 2 diabetes (T2D). Methods: We performed a secondary analysis of cross-sectional data from youth (55% African American, 70% female) ages 10–23 years with T2D (n = 90), obesity (BMI > 95th; n = 128), or healthy weight (BMI < 85th; n = 197). Whole body (less head) areal bone mineral density (aBMD) was assessed by dual-energy X-ray absorptiometry (DXA). aBMD was expressed as age-, sex-, and ancestry-specific standard deviation scores (Z-scores). Whole body aBMD Z-scores were adjusted for height-for-age Z-score. Diet was assessed via three-day diaries, and the Healthy Eating Index (HEI) was computed. Total HEI score and HEI subcomponent scores were compared across groups, and associations with aBMD Z-scores were assessed via linear regression adjusted for group, age, sex, and ancestry. Results: Mean HEI was similar between the healthy weight, obesity, and T2D groups. Several HEI sub-components differed between groups, including meats and beans, total vegetables, milk, saturated fat, sodium, oils, and empty calories. The obesity and T2D group had significantly greater aBMD Z-scores compared to the healthy weight group. Multiple linear regression analyses revealed a significant positive association between HEI and aBMD Z-score (*p* < 0.05). The HEI sub-components for whole grains (*p* = 0.052) and empty calories (*p* < 0.05) were positively associated with aBMD Z-score. Conclusions: Individuals that followed a dietary pattern more closely aligned with the Dietary Guidelines for Americans had greater bone density. Since few studies have investigated the role of diet on bone in youth with obesity-related conditions, additional research is required among these populations.

## 1. Introduction

A recent National Osteoporosis Foundation (NOF)-sponsored position statement on nutritional determinants of peak bone mass summarized the clinical data relating to effects of various diet constituents on childhood and adolescent bone accrual [1]. The authors of this report concluded that the strongest evidence supporting a bone-related benefit was for calcium, vitamin D, and dairy, but due to a limited number of studies in younger individuals, there was only modest evidence supporting an effect of diet patterns, such as those characterized by high consumption of fiber, fruits, and vegetables, on peak bone mass. Accordingly, there is a need for clinical studies evaluating associations between the quality of the overall diet and bone outcomes during the years surrounding peak bone mass.

The Dietary Guidelines for Americans (DGA) outlines national recommendations for dietary intake to promote overall health and reduce chronic disease risk [2]. Since the DGA encompasses various individual nutrients and food groups, the Healthy Eating Index (HEI) is a scoring system intended to reflect the quality of an individual and/or population’s diet pattern with respect to key guidelines set forth in the DGA [3]. The first iteration of the HEI was specific to guidelines from the 1995 DGA Report, and was subsequently updated to reflect the 2005 DGA, including scoring subcomponents for nutrients and food groups that should be consumed either in “adequate” (e.g., total fruit and vegetables, total and whole grains, milk, oils) or “moderate” (saturated fat, sodium, and “empty” calories) amounts [3]. Results from the National Health and Nutrition Examination Survey (NHANES) suggest that diet quality assessed via HEI varies across the lifespan and is lowest during childhood and adolescence [2], which reflects poorer alignment with DGA guidelines. Since the majority of the adult skeleton is accrued before the age of 20 years [4], suboptimal diet quality during the pivotal childhood and adolescent years might threaten peak bone mass attainment. Studies in adults suggest that consuming a diet that is more closely aligned with guidelines set forth in the DGA might be associated with lower risk for fracture [5]. The vast majority of studies evaluating associations between diet patterns and bone in youth employed “data-driven” methods, such as cluster or factor analysis, and have not focused on the HEI [6,7].

Children with obesity [8] and adults with type 2 diabetes (T2D) [9] are at increased risk for fracture, but youth with obesity and related chronic health conditions are often excluded from studies involving diet and bone. In a recent study from the current cohort, we reported a potential adverse influence of T2D on bone density in adolescents and young adults, and that dietary calcium intake and vitamin D status were lower in youth with obesity and T2D compared to peers with healthy body weight [10]. Since diet quality contributes to obesity-related chronic disease risk and plays a pivotal role in peak bone mass attainment [1,11], additional studies in actively growing individuals with obesity and related chronic health conditions are required.

We present a hypothesis generating study aimed at assessing relationships between diet quality, measured via HEI, and areal bone mineral density (aBMD) in a cross-sectional sample of youth with healthy weight, obesity, and T2D. Additional analyses were performed in relation to individual subcomponents of the HEI and aBMD.

## 2. Materials and Methods

This study was a secondary analysis of previously collected cross-sectional data including African American and non-African American males and females, ages 10–23 years with T2D, obesity, or healthy weight [9,11,12]. Participants in the healthy weight group had a body mass index (BMI)-for-age between the 5th and 85th percentile, and participants in the obese group had a BMI-for-age greater than the 95th. Diagnosis of T2D was based on American Diabetes Association criteria [13], and all subjects with T2D were negative for glutamic acid decarboxylase, islet cell autoantigen-512, and insulin autoantibodies. Individuals from the healthy weight and obese groups with HbA1c greater than 6.5% or fasting plasma glucose greater than 126 mg/dL were not included in this study. Written informed consent/assent was obtained from each subject/guardian. The Institutional Review Board for Human Subjects at Cincinnati Children’s Hospital Medical Center approved all study protocols and procedures.

### 2.1. Anthropometrics

While subjects were shoeless and wearing light indoor clothing, height was measured using a wall-mounted stadiometer (Veeder-Rood, Elizabethtown, NC, USA) and weight was measured using an electronic scale (Health-O-Meter). Standard deviation scores (“Z-scores”) for height and BMI were calculated [14]. For subjects greater than 20 years of age, an age of 20 years was used for all anthropometric (and bone density) Z-score calculations.

### 2.2. Dual-Energy X-ray Absorptiometry

Whole body (less head) aBMD (g/cm^2^) was assessed via dual-energy X-ray absorptiometry (DXA; Hologic QDR 4500A, Hologic, Inc., Bedford, MA, USA), and all scans were analyzed using Apex software (version 5.5.3). Age-, sex-, and population ancestry-specific standard deviation scores, or “Z-scores,” for aBMD were calculated using published pediatric growth charts [15]. aBMD Z-scores were subsequently adjusted for height Z-score using a previously published method [16].

### 2.3. Diet Intake

Diet intake was estimated using self-administered 3-day records, which were subsequently analyzed using the Nutrition Data System for Research software. Since data collection for this study was conducted from 2005 to 2009, the HEI-2005 was calculated. The HEI-2005 is comprised of 12 individual sub-components, including 9 “adequacy” components and 3 “moderation” components [3,17]. The adequacy components include total fruit, whole fruit, total vegetables, dark green and orange vegetables and legumes, total grains, milk, meat and beans, and oils, and the moderation components include saturated fat, sodium, and empty calories (calories from solid fats, alcoholic beverages, and added sugars). Total HEI score can range from 0 to 100, with higher scores being more closely aligned with DGA guidelines and therefore considered “healthier”.

### 2.4. Statistical Analysis

Between-group comparisons for continuous variables were assessed using analysis of variance or Kruskal–Wallis/Dunn’s tests (with Bonferroni post-hoc adjustment) and comparisons of categorical variables were assessed using Pearson’s chi square test. Estimated energy requirements, calculated using equations published by the Institute of Medicine [18], were computed. Subjects that reported a total caloric intake less than 75% of that required for energy maintenance were excluded from this study.

The relationship between HEI and aBMD Z-score was assessed using multiple linear regression while accounting for age, sex, ancestry, and group. A HEI by group interaction term was included to determine whether the association between HEI and BMD differed between youth with healthy weight, obesity, and T2D. Additional regression analyses were performed for each of the 12 individual HEI sub-components. Scores for the meats/beans and total grains HEI sub-component scores tended to truncate toward the maximum score of 10 and 5, respectively. For this reason, analyses used a group variable for meats/beans sub-component scores less than 10 or equal to 10, and for the total grains sub-component score, less than 5 or equal to 5. Sensitivity analyses were performed in the healthy weight and obesity/T2D groups separately. For all analyses, the distribution of regression residuals was inspected for normality. Finally, the unadjusted association between HEI and aBMD Z-score was assessed using Pearson’s correlation.

All statistical analyses were performed using STATA (version 15.1). *p*-values < 0.05 were considered statistically significant.

## 3. Results

Compared to the healthy weight group (11%), a greater proportion of study subjects in the obesity (38%) and T2D (41%) groups were excluded from this study due to likely under-reporting of diet intake (energy intake less than 75% of estimated energy requirement). In the healthy weight, obesity, and T2D groups, HEI scores did not differ between the included and excluded study subjects (all *p* = 0.23 to 0.54).

Descriptive characteristics of our study sample are presented in Table 1. Our study sample included a greater proportion of females versus males and African Americans versus non-African Americans. Briefly, there were significant differences in ancestry, BMI Z-score, aBMD Z-score, and HbA1c between the healthy weight, obesity, and T2D groups (all *p* < 0.05). Total HEI score and several HEI sub-component scores (meat and beans, total vegetables, milk, saturated fat, sodium, oils, and empty calories) differed between youth with healthy weight, obesity, and T2D (Table 2). There was a significantly greater proportion of subjects in the T2D group (71%) with a meats/beans sub-component score of 10 compared to the healthy weight (49%) and obesity (50%) groups (*p* = 0.001).

**Figure 1:**
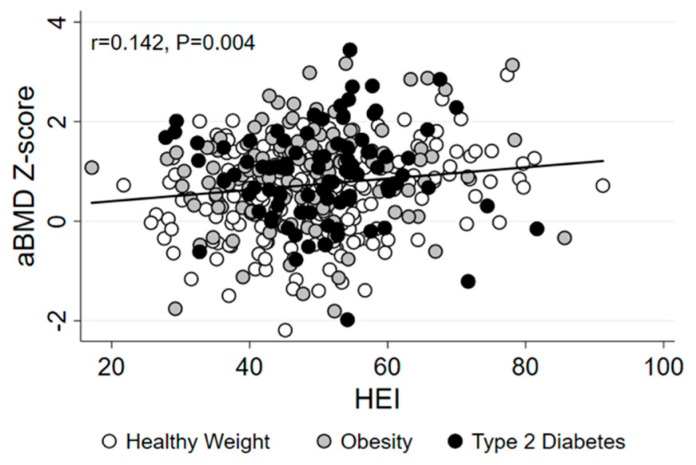
Scatter plot presenting the bivariate association between HEI and aBMD Z-score in youth with healthy weight (white dots), obesity (gray dots), and T2D (black dots). HEI, healthy eating index; aBMD, areal bone mineral density.

To determine whether the association between HEI and aBMD differed between youth with healthy weight, obesity, and T2D, we first tested whether there was a significant interaction between HEI and group with respect to aBMD Z-score. There was no evidence of an interaction between HEI and group (*p* = 0.362), so main analyses were performed in the healthy weight, obesity, and T2D groups combined. The bivariate association between HEI and aBMD Z-score was positive and statistically significant (Figure 1), and HEI remained significantly associated with aBMD Z-score after adjusting for age, sex, ancestry, and group (Table 3). The empty calorie HEI sub-component score was significantly positively associated with aBMD Z-score. The HEI sub-component for whole grains was also positively associated with aBMD Z-score, but this association was not statistically significant (*p* = 0.052). Remaining HEI sub-components were not associated with aBMD. Obesity and T2D groups were associated with increased aBMD in all analyses. African American ancestry was associated with lower aBMD in all analyses, except for the model including total HEI. Further, female sex was associated with increased aBMD in the model including the meats and beans sub-component score (all *p* < 0.05).

Since a significantly lower proportion of study subjects from the healthy weight group compared to the obesity and T2D groups were excluded from this study due to potential misreporting of diet intake, sensitivity analyses were performed in the healthy weight and obesity/T2D groups separately while accounting for age, sex, and ancestry. In the healthy weight group, total HEI score (b = 0.012, SE = 0.005, Beta = 0.175, *p* = 0.023) and the whole grains HEI sub-component score (b = 0.097, SE = 0.039, Beta = 0.187, *p* = 0.015) were significantly positively associated with aBMD Z-score. The empty calories HEI sub-component score was positively associated with aBMD Z-score, but this relationship was not statistically significant (b = 0.023, SE = 0.012, Beta = 0.143, *p* = 0.054). Total HEI and sub-component scores were not significantly associated with aBMD Z-score in the obesity and T2D group.

## 4. Discussion

Diet is an important modifiable determinant of bone health [1], and studies in adults support a potential benefit of select nutrients, food groups, and diet patterns on bone density and fracture [5]. Few studies have assessed associations between diet quality and bone density during critical periods of growth, particularly in youth with obesity and related chronic health conditions. The current study addressed this research gap by examining associations between the HEI, a measure of diet quality, and bone density in a cross-sectional sample of youth with healthy weight, obesity, and T2D. We report a significant positive association between HEI and aBMD, suggesting that youth reporting a diet pattern more closely aligned with DGA guidelines tended to have greater aBMD Z-scores. Secondary analyses focusing on individual HEI sub-components identified lower consumption of empty calories and higher consumption of whole grains as positive correlates of aBMD. Since adolescents and young adults tend to under-consume whole grains and over-consume empty calories [2], these HEI sub-components represent potential targets for improvement of diet quality to help promote optimal peak bone mass attainment.

Diet is a modifiable contributor to overall health status, but the majority of Americans do not meet recommended intake amounts for most food groups and essential nutrients [2]. With respect to peak bone mass, several dietary factors have a favorable effect on bone accrual during the growing years. Studies focused on a single nutrient and/or food group have generally demonstrated a favorable effect of calcium, vitamin D, and dairy on bone [1]. However, since diet is comprised of combinations of nutrients and bioactives that are highly correlated and interact with one another, studies focused on a single nutrient and/or food group have limited translation. To account for the quality of the overall diet, the current study focused on the HEI, which is a composite score indicating how closely an individual’s and/or population’s diet aligns with the recommendations outlined in the DGA [12]. The main finding from this study was that individuals that reported consuming a diet more closely aligned with national guidelines (higher HEI score) tended to have greater bone density compared to those with lower adherence (lower HEI score). Observational and intervention studies suggest a favorable effect of other diet patterns, such as the Dietary Approaches to Stop Hypertension (DASH) or Mediterranean Diet, on bone outcomes in adults and children [5,6,13]. Studies involving the HEI have primarily been confined to adults and suggest a potential benefit with respect to fracture risk [5,14]. The current study is the first to report these associations with respect to aBMD in youth.

The total HEI score is comprised of 12 individual sub-components for key food and nutrient recommendations from the DGA report. Two individual HEI sub-components were positively associated with aBMD Z-scores, including the empty calories and whole grains sub-components. Although there were slight differences between the three groups, most subjects reported relatively high consumption of protein-rich foods. Nationally representative data from NHANES indicate that most Americans meet or exceed intake recommendations for daily protein consumption [2]. Studies in adults assessing the effect of protein intake on bone have yielded inconsistent findings, but meta-analyses evaluating prospective and intervention studies suggest a likely positive effect on bone [15,16]. Randomized interventions involving bone augmenting effects of protein during the growing years are sparse.

In contrast to protein, most Americans fail to meet intake recommendations for whole grains [2]. In our study, the whole grains HEI sub-component score was considerably low regardless of group. Nevertheless, we observed positive associations between whole grain consumption and aBMD Z-score. The whole grains sub-component was only marginally positively associated with aBMD Z-score in the total cohort, but this association was statistically significant within the healthy weight group only. Whole grains are a primary source of dietary fiber, which has been posited to benefit bone accrual, likely mediated through modification of the gut microbiome and subsequent increases in mineral absorption [17,18,19]. Increased whole grain consumption is also associated with decreased risk of T2D in adults [20], perhaps due to the effects of fiber on glycemic control [21]. Thus, whole grains might represent a potential dietary target for improving both metabolic and skeletal health among youth at risk for diabetes. We also report greater aBMD Z-scores in individuals with lower consumption of foods in the “empty calories” HEI sub-component, which includes calories from solid fats, alcoholic beverages, and added sugar. Compared to other HEI sub-components, the empty calorie sub-component is most closely reflective of total diet quality [3], which might help explain the similar associations with total HEI score and aBMD.

Inclusion of youth with obesity and T2D was a unique feature of this study. Despite the concerning trends in childhood obesity [22] and youth-onset T2D [23], individuals with obesity and related health conditions are often excluded from studies involving bone health. One potential explanation for the paucity of data relating to diet and bone in youth with obesity might involve heightened concerns of misreporting diet intake. To address this concern, we excluded subjects that were suspected to have misreported energy intake based on expected energy needs for weight maintenance. A greater percentage of youth with obesity and T2D were excluded from this study due to potential misreporting of energy intake, highlighting challenges and concerns of self-reported diet intake methodologies in youth with obesity and related diseases. Considering these differences, we performed sensitivity analyses in the healthy weight and obesity/T2D groups separately. There were overall null associations between HEI and aBMD in analyses including only the obesity and T2D groups. We suspect that this was attributed to greater error in self-reported diet intake among youth with obesity and T2D rather than a less pronounced biological effect of diet on aBMD. In contrast, results in the healthy weight group generally reflected those reported in the total cohort, suggesting that findings in our main analyses were driven by the healthy weight group.

The cross-sectional design of this study is a key limitation. Prospective observational studies and randomized controlled trials are required to evaluate the bone augmenting effects of diet on peak bone mass more thoroughly. The current study was limited to aBMD assessed at the whole body region, which is a skeletal site that is representative of cortical bone and is recommended by the ISCD for pediatric clinical DXA evaluations [24]. Since skeletal regions comprised of mostly trabecular bone, such as the lumbar spine or distal forearm, were not examined in this study, it is unknown whether HEI is associated with trabecular bone density. Earlier studies in children and adolescents reported positive associations between dietary fiber consumption and ultradistal forearm BMD from DXA [25], as well as a potentially favorable effect of dairy consumption on distal radius volumetric BMD from peripheral quantitative computed tomography (pQCT) [26]. Future studies should evaluate both the cortical and trabecular bone compartments using imaging methodologies that are translatable to the clinical arena (i.e., DXA), as well as those methodologies that can evaluate characteristics of bone volumetric density and strength (i.e., high resolution pQCT). More thorough measures of self-reported diet intake and objectively measured dietary biomarkers should be considered in future studies.

## 5. Conclusions

A recent NOF-sponsored position statement on nutritional determinants of peak bone mass emphasized the importance of achieving federal dietary guidelines for attainment of peak bone mass [1]. Results from the current study align with this recommendation, as youth that reported consuming a diet pattern that was more closely aligned with key recommendations set forth in the DGA was associated with greater aBMD. Future studies should evaluate bone outcomes prospectively during critical periods of growth while considering subjective measures of diet quality and objective biomarkers of nutrient and food group consumption to more adequately identify dietary contributors to peak bone mass attainment.

## Figures and Tables

**Table 1:** Subject characteristics.

	Healthy Weight	Obesity	Type 2 Diabetes	*p*
	n = 197	n = 128	n = 90	
Age (years)	17.8 (3.6)	18.5 (3.4)	18.5 (3.0)	0.089
Sex (% female)	65.6%	76.4%	71.0%	0.087
Ancestry (% African American)	50.0%	66.2%	51.4%	0.006
Height Z-score	0.15 (0.99)	0.20 (1.10)	0.24 (1.17)	0.767
BMI Z-score	0.14 (−0.43 to 0.52)	2.33 (1.73 to 2.90) ^a^	1.89 (1.47 to 2.84) ^a^	<0.001
aBMD Z-score	0.53 (0.85)	0.84 (0.93) ^a^	0.92 (0.88) ^a^	<0.001
Energy (kcal/day)	2305 (802)	2351 (717)	2263 (798)	0.702
HbA1c (%)	5.3 (5.1 to 5.5)	5.5 (5.2 to 5.7) ^a^	6.9 (5.7 to 10.2) ^a,b^	<0.001

Values are mean (standard deviation) or median (interquartile range). ^a^ Significantly different than healthy weight, ^b^ Significantly different than obese. BMI, body mass index; aBMD, areal bone mineral density; HbA1c, hemoglobin A1c.

**Table 2:** HEI and HEI sub-components in youth with healthy weight, obesity, and T2D.

	Maximum Score	Healthy Weight	Obesity	T2D	*p*
Total HEI	100	49.7 (41.6 to 56.4)	46.6 (39.9 to 52.3)	51.1 (44.1 to 55.6) ^b^	0.024
HEI Sub-Components					
Adequacy Sub-Components					
Meats and beans	10	9.9 (6.4 to 10)	10.0 (7.09 to 10)	10.0 (9.3 to 10) ^a,b^	0.001
Total vegetables	5	2.3 (1.4)	2.1 (1.2)	2.7 (1.4) ^a,b^	0.004
Dark green and orange vegetables	5	1.1 (1.6)	1.0 (1.5)	1.2 (1.7)	0.563
Total fruit	5	1.1 (0.0 to 2.5)	0.7 (0.0 to 2.2)	0.6 (0.0 to 2.2)	0.166
Whole fruit	5	0.1 (0.0 to 2.2)	0.0 (0.0 to 1.1)	0.0 (0.0 to 0.9) ^a^	0.080
Total grains	5	4.7 (0.6)	4.7 (0.6)	4.7 (0.6)	0.844
Whole grains	5	1.1 (0.0 to 2.5)	0.6 (0.0 to 2.0)	0.8 (0.0 to 1.5)	0.120
Milk	10	6.2 (2.8)	5.1 (2.8) ^a^	5.3 (2.6)	0.002
Oils	10	7.2 (2.7)	7.3 (2.8)	8.3 (2.3) ^a,b^	0.003
Moderation Sub-Components					
Saturated fat	10	4.9 (3.1)	5.0 (3.0)	3.8 (3.1) ^a,b^	0.008
Sodium	10	2.6 (2.3)	2.8 (2.4)	1.3 (1.9) ^a,b^	<0.001
Empty calories	20	8.4 (5.3)	7.5 (5.5)	11.0 (5.3) ^a,b^	<0.001

^a^ Significantly different than healthy weight, ^b^ Significantly different than obese. HEI, Healthy Eating Index.

**Table 3:** Relationships between HEI and HEI sub-components with aBMD Z-score in youth with healthy weight, obesity, and T2D combined.

	b	SE	Beta	*p*
Total HEI	0.009	0.004	0.114	0.025
HEI Sub-Components				
Adequacy				
Meats and beans	0.138	0.092	0.074	0.133
Total vegetables	0.022	0.034	0.032	0.520
Dark green and orange vegetables	0.022	0.028	0.038	0.438
Total fruit	0.018	0.029	0.031	0.528
Whole fruit	0.021	0.030	0.036	0.487
Total grains	−0.022	0.095	0.011	0.817
Whole grains	0.059	0.030	0.098	0.052
Milk	0.013	0.017	0.040	0.439
Oils	0.007	0.017	0.021	0.666
Moderation				
Saturated fat	0.004	0.014	0.012	0.806
Sodium	−0.032	0.020	−0.081	0.103
Empty calories	0.022	0.008	0.131	0.010

All analyses include sex, age, ancestry, and group. b, unstandardized regression coefficient; SE, standard error; Beta, standardized regression coefficient.

## Data Availability

Available upon request.
